# Massive Thrombosis of the Right Atrium Extended to the Superior Vena Cava at the Diagnosis of Acute Myeloid Leukemia

**DOI:** 10.1155/2016/6802429

**Published:** 2016-10-26

**Authors:** Bienvenu Houssou, Gnon Gourou Orou-Guiwa, Rachida Habbal, Meryem Qachouh, Asmaa Quessar

**Affiliations:** ^1^Department of Clinical Hematology and Pediatric Oncology, Hospital August 20, 1953, University Hassan II, Casablanca, Morocco; ^2^Department of Cardiology, Ibn Rochd Hospital, University Hassan II, Casablanca, Morocco

## Abstract

*Introduction*. Venous thromboembolic disease is a common complication found in 8% of patients with acute myeloid leukemia. The location at the right atrium is exceptional. These last fifty years, only 6 cases of thrombosis of the atrium in the diagnosis of acute myeloid leukemia were published on PubMed search engine.* Case Presentation*. 35-year-old farmer, who had been admitted by emergency department for superior vena cava syndrome and had a hyperleukocytic AML with complex karyotype associated with a significant thrombosis of the right atrium, extended all along the superior vena cava. He has been treated by the 2011 AML protocol using low molecular weight heparin and died from respiratory distress.* Conclusions*. If thrombosis is common in AML, the location in right atrium is rare. Its management requires surgery that is sometimes difficult to achieve.

## 1. Introduction

Venous thromboembolism (VTE) is a frequent complication found in approximately 11% of patients with acute leukemia [1]. It is associated in 13.5% with the presence of a central venous catheter, or the steroids use, asparagine in acute lymphoblastic leukemia or inherited thrombophilic abnormalities [2]. It is found in approximately 8% of acute myeloid leukemia (AML) patients [1] and sits in 73.3% of cases in the deep veins of the upper limbs: axillary vein under subclavian and jugular veins and 16% in the deep veins of the lower limbs: femoral vein, superficial femoral, popliteal, and iliac veins [3]. Pulmonary embolism represents 7.8% of the locations and is potentially fatal [3]. Intracardiac thrombosis localizations in AML are rare and scarcely reported in the literature.

Using keywords thrombosis, atrium, and leukemia for research on PubMed there are only 6 cases of thrombosis of the right atrium which were published in the last fifty years.

We report a rare case of a AML revealed by a superior vena cava syndrome secondary to thrombosis of the right atrium extended all along the superior vena cava.

## 2. Medical Observation

**Figure 1:**
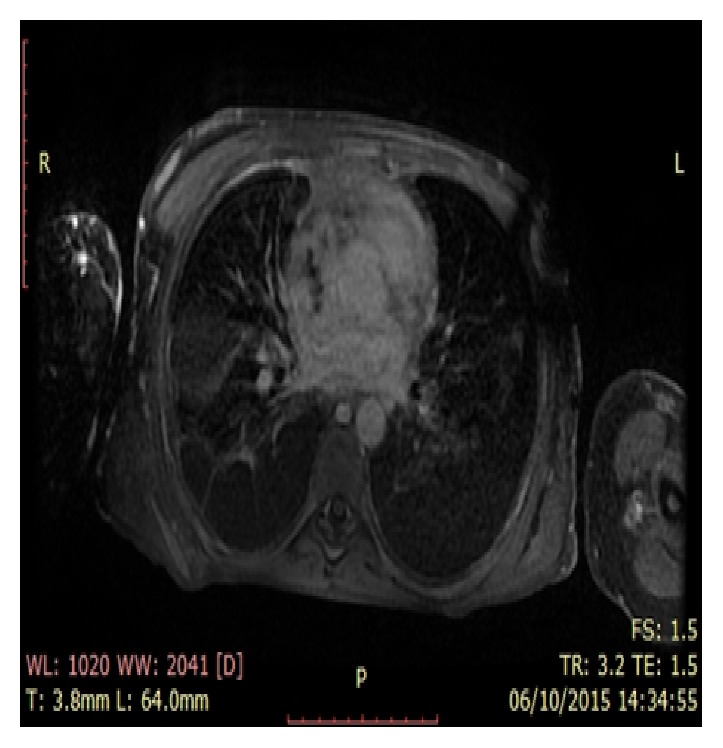
Cardiac MRI, thrombosis superior vena cava. Cross section of the cardiac MRI through the superior vena cava, the white ring shows thrombosis in the superior vena cava.

**Figure 2:**
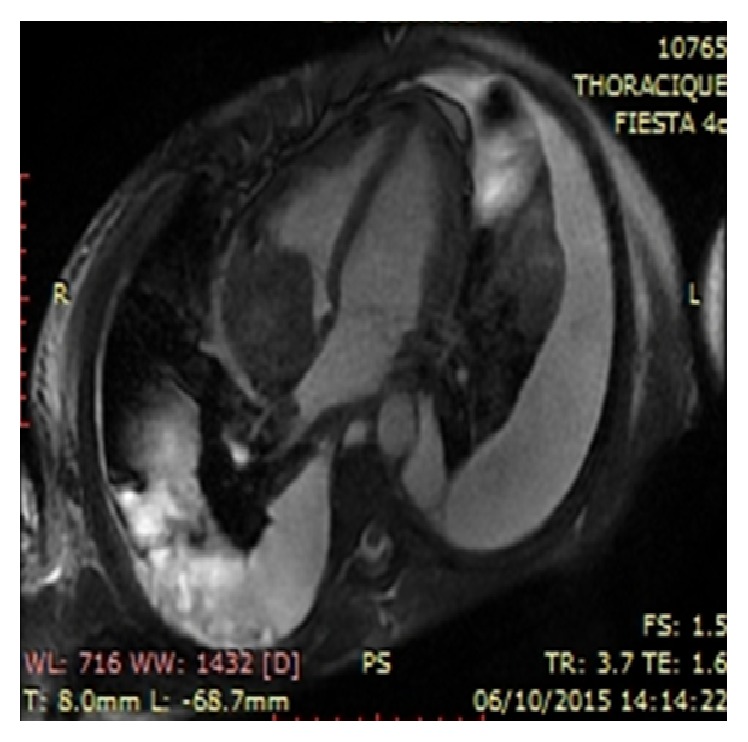
Cardiac MRI, thrombosis occupying most of the right atrium. Cross section of the cardiac MRI through the atria, the white ring shows the massive thrombosis at the right atrium.

35-year-old man, active farmer, without past medical history, had been admitted to emergency department for superior vena cava syndrome (SVCS). Complete blood count (CBC) had revealed hyperleukocytosis with peripheral blasts, white blood cell count: 116 G/L with 95% blasts and platelets 212 G/L, and hemoglobin rate: 13.1 g/dL. Chest X-ray had revealed a pleural effusion syndrome of mean volume at left side and of great abundance at right side, with a right basal pulmonary condensation syndrome. He had been sent to the clinical hematology unit for treatment. The anamnesis revealed that the symptoms had begun 10 days before the admission by gradual swelling of the neck extended to the face with dyspnea all occurring in a context of impaired general condition. Physical examination revealed an altered condition with WHO: 3, temperature: 37° 5C, blood pressure: 12/7 cmHg, SaO2 82% on room air, cardiac frequency: 84 bpm, weight: 52 kg, height: 1.72 m, a SVCS, a bilateral pleural effusion syndrome, and right pulmonary condensation syndrome. Bone marrow aspiration had concluded in 85% large blasts sizes with irregular nucleus, nucleolus, hypogranulous cytoplasm, myeloperoxidase negative, and concluded in probable monocytic AML (AML5). Immunophenotyping concluded in acute myeloid leukemia: MPO: 75%, CD117: 98%, CD38: 98%, CD33: 26%, CD13: 22%, CD11c: 5%, HLA-DR: 3%, and CD14: 0%. Lymphoid T and B markers were negative. The karyotype revealed a complex hyperploidy: 51, XY, +4, +8, del (11) (q23), 19, 21, 21, 22 [17]/46, and XY [9]. To explore the SVCS, electrocardiography was normal, echocardiography revealed dilated right atrium containing a pedunculated mass of 25.6 cm^2^ and heterogeneous irregular surface, appended to the atrial face of the anterior tricuspid valve with subtotal obstruction of the tricuspid orifice; the systolic ejection fraction of left ventricle is 60%. Systolic pulmonary artery pressure was 45 mmHg; he had moderate pulmonary hypertension. Thoracic angio-TDM showed intracardiac mass with a large parietal base, filling almost all the right atrium, penetrating the right ventricle, and surrounding the orifice of the superior vena cava which is not opacified. The mass dimension is 51.6 × 59 mm, condensation image site in the posterior part of the upper lobe of the right lung surrounded by a frosted glass image. The nature of this mass was unknown and cardiac magnetic resonance imaging had been objectified: venous thrombosis extended to the full height of the superior vena cava with thrombosis of the right atrium (Figures 1 and 2). To explore this thrombosis, tests of hemostasis are as follows: prothrombin 45%, partial thromboplastin time enhancer: 24.5 s (normal: 25 ± 5 s), fibrinogen: 0.90 g/L, and D-dimers: 6000 mg/mL. A thrombophilic factor V Leiden and the G20210A mutation of thrombin were negative. The proteins C and S and antithrombin III were normal.

Moreover, the fluid and electrolyte balance and infectious workup were normal.

In all, this is a case of a 35-year-old patient with hyperleukocytic AML 5 with complex karyotype complicated by a massive thrombosis extended from the atrium to the superior vena cava.

He has been hospitalized and received hydration, cytarabine 100 mg flash for a 4 days' cytoreduction, rasburicase 0.2 mg/kg/24 h the first two days, enoxaparin 10,000 IU per day, ciprofloxacin 200 mg/8 h, and fluconazole 200 mg/12 h. On the 5th day of hospitalization, he was induced to AML protocol: cytarabine 100 mg/m^2^/12 h of J1 to J10 and daunorubicin 50 mg/m^2^ once daily D2, D4, and D6. The electrolytic test has been done twice daily and the blood count once a day, and diuresis was assessed every 4 hours.

A multidisciplinary staff had been held between hematologists, cardiologists, and cardiovascular surgeons and it has been decided not to perform surgery in view of the bleeding risk and to continue the anticoagulation treatment.

The evolution was marked on the 8th day of induction by aplasia with a hemoglobin rate of 6 g/dL, white blood cells: 0.1 G/L with neutrophils: 0.02 g/L, and platelets: 20 G/L for which he has been transfused by red blood cells and platelets and then an accentuation of dyspnea, edema of the face and neck down to the chest and upper extremities, and the appearance of collateral venous circulation in the chest and a recurrent left pleural effusion after each puncture. In the 12th day of treatment, he died in a respiratory distress syndrome context.

## 3. Discussion

The patient had two emergencies: hyperleukocytic AML associated with massive thrombosis of the right atrium extending to the superior vena cava.

Hyperleukocytic AML represents 10 to 18% of the adult AML [4]. This is an emergency diagnosis due to the risk of hyperviscosity syndrome resulting in neurological signs (confusion, psychomotor slowing, and blurred vision) of leukostasis mainly with respiratory signs (dyspnea and acute respiratory distress syndrome); coagulopathy (DIC), and tumor lysis syndrome [5].

Thrombosis observed in AML is the result of multiple mechanisms: the release of procoagulant particles; reduced levels of natural anticoagulants; the decrease in the fibrinolytic activity; increased coagulation factors; platelet activation; and cytokine-induced expression of tissue factor in endothelial cells [6]. Acute promyelocytic leukemia is the most common AML provider of VTE. The AML 5 is associated in 25% of patients with thrombosis.

Thrombosis in AML preferentially seats in the upper and lower limbs and the lungs [3]. Generally, the thrombus formation is less frequent in the right atrium compared with the left atrium [7]. It occurs in the presence of implanted catheters, particularly at the junction of the superior vena cava and the right atrium and in patients with atrial fibrillation or prothrombotic states [7]. Thoracic angio-TDM is useful in diagnosis; magnetic resonance imaging can be used in patients in which radio-contrast agents are contraindicated [8]. Thoracic angio-TDM could not afford to know the nature of the right atrial mass of the patient and it is the magnetic resonance imaging that made the diagnosis of right atrial thrombosis. The treatment is not codified but several studies report a percutaneous stent implantation in patients with severe respiratory distress symptoms before the etiological treatment improves prognosis [9]. During evolution, the observed venous circulations are due to the fact that, because of this thrombosis, the body uses the ways of substitutions made by the azygos system, lumbar veins, spinal plexus, and thyroid veins which result in appearance of edema of neck, chest, and limbs [1]. Pulmonary hypertension could be an exaggerated cause of respiratory distress.

## 4. Conclusions

If thrombosis is common in AML, the location in right atrium is rare, and one should think about it before a superior vena cava syndrome. Its management requires surgery that is sometimes difficult.

## Abbreviations

VTE: Venous thromboembolism
AML: Acute myeloid leukemia
SCVS: Superior vena cava syndrome
CBC: Complete blood count.
